# Tocilizumab combined with cyclophosphamide for the treatment of rapidly progressive refractory systemic sclerosis with predominant cardiac involvement: a case report

**DOI:** 10.3389/fimmu.2025.1614714

**Published:** 2025-07-03

**Authors:** Zhong-Chao Fu, Jing Ji, Xiao-Gui Cheng, Ran-Geng Shi, Xiao-Feng Mo, Zhi-Zhu Yang

**Affiliations:** ^1^ Department of Rheumatology and Immunology, The Third Affiliated Hospital of Shenzhen University, Shenzhen, Guangdong, China; ^2^ Department of Pharmacy, The Third Affiliated Hospital of Shenzhen University, Shenzhen, Guangdong, China

**Keywords:** systemic sclerosis, cardiac involvement, tocilizumab, cyclophosphamide, combination therapy

## Abstract

Systemic sclerosis (SSc) with multi-organ involvement poses significant therapeutic challenges. We present a case of rapidly progressive, refractory SSc with cardiac, musculoskeletal, and skin manifestations that was successfully managed with a combination of cyclophosphamide (CYC) and tocilizumab (TCZ). In this case, a 25-year-old female patient with rapidly progressive SSc developed severe skin sclerosis (mRSS 46), myofascial edema, myopathy (CK 923 U/L), joint flexion contractures, and pericardial effusion. Initial therapy with glucocorticoids and CYC showed limited efficacy. After therapeutic escalation to an alternating 4-week regimen (TCZ 8 mg/kg and CYC 600 mg administered sequentially every 2 weeks), pericardial effusion resolved completely, skin softening was observed (mRSS reduced to 32), and functional status improved significantly, with no significant adverse events reported. This case highlights the potential efficacy and safety of the CYC-TCZ combination therapy for refractory SSc, particularly in patients with cardiac involvement. These findings support the need for further exploration of this regimen in clinical trials.

## 1 Introduction

Systemic sclerosis (SSc) is a complex autoimmune disease characterized by immune dysregulation, fibrosis, and vasculopathy. Cardiac involvement (e.g., pericardial effusion) and myopathic degeneration represent high-risk disease phenotypes, frequently exhibiting resistance to first-line immunosuppressants. Patients with visceral involvement have a poor prognosis and high mortality rates (1). Although cyclophosphamide (CYC) and tocilizumab (TCZ) have been individually used in the treatment of SSc (1–5), their combination remains understudied. Nevertheless, this combination may offer synergistic benefits for refractory cases. We present a case of rapidly progressive and refractory SSc with cardiac involvement that was successfully treated with CYC-TCZ, highlighting the therapeutic rationale and safety profile of this approach.

## 2 Case report

A 25-year-old female presented with swelling and pain in the hands in February 2023, followed by Raynaud’s phenomenon in May 2023. Three months later, her condition worsened. Laboratory tests in August 2023 revealed positive serum anti-Scl-70 antibodies (moderate titer) and anti-nuclear antibody (ANA) with a titer of 1:640. She was diagnosed with SSc and treated with methotrexate (MTX) and traditional Chinese medicine, which showed limited therapeutic efficacy. By December 2023, she developed progressive severe skin sclerosis and musculoskeletal involvement, including bilateral thigh pain, joint tenderness, and restricted mobility, resulting in prolonged bed rest and a 11-kg weight loss (from 53 kg to 42 kg) over this period, attributed to malabsorption. Given disease progression and diminished treatment confidence, she temporarily discontinued therapy. In February 2024, she experienced new-onset dyspnea and anorexia without fever. Following psychological counseling and medical guidance, she was admitted to the hospital in March 2024.

**Figure 1:**
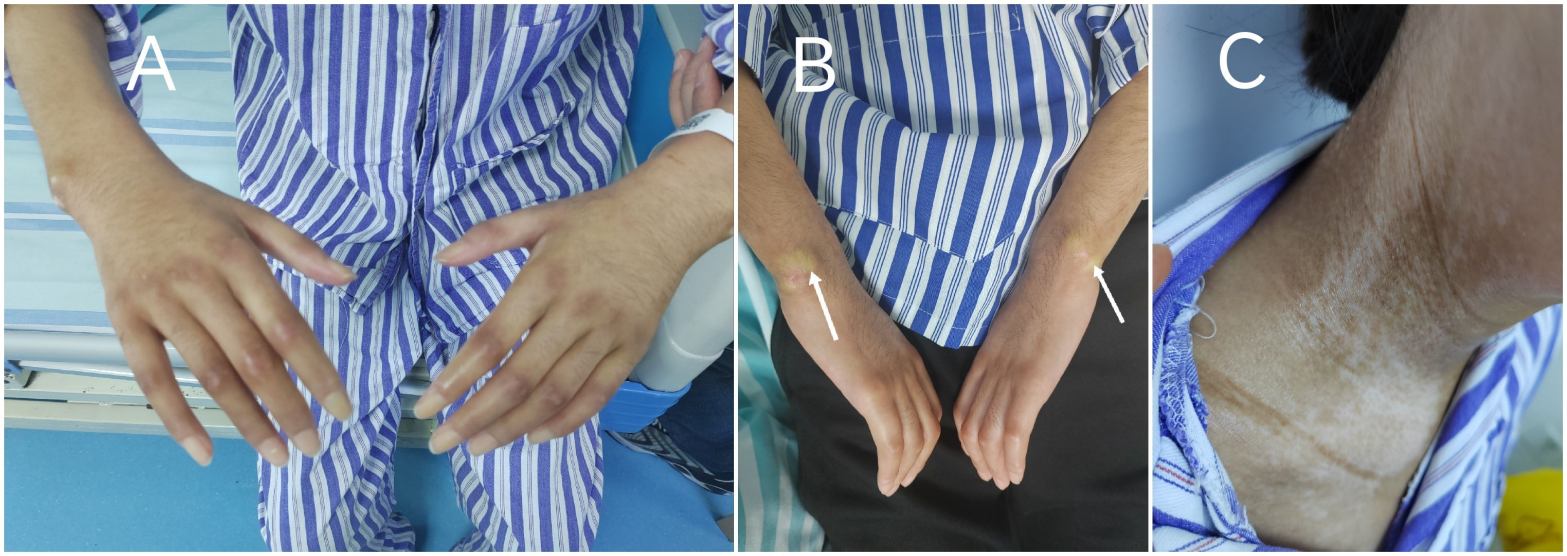
Characteristic cutaneous manifestations of systemic sclerosis on admission: **(A)** Diffuse cutaneous sclerosis with shiny appearance and restricted joint mobility over the upper extremities. **(B)** Subcutaneous calcinosis at the bilateral wrist extensor surfaces. **(C)** “Salt-and-pepper” dyschromia on the neck.

Physical examination revealed a body mass index (BMI) of 15.4 kg/m². She presented with diffuse severe skin sclerosis, characterized by shiny, indurated skin involving both lower and upper limbs (**Figure 1A**), face, neck, and chest, along with subcutaneous calcinosis at the bilateral wrists (**Figure 1B**). Notable cutaneous features included a “mask-like” facies and “salt-and-pepper” skin changes (**Figure 1C**). The modified Rodnan skin score (mRSS) was 46. Musculoskeletal evaluation demonstrated joint tenderness in the knees, ankles, metacarpophalangeal joints, and lumbosacral spine, accompanied by proximal muscle weakness with Medical Research Council (MRC) grade 4 in both upper and lower limbs. Passive range of motion was absent in the knees and wrists, rendering her non-ambulatory. The nursing team documented an Activities of Daily Living (ADL) score of 35/100. Peripheral venous access via the basilic vein was unfeasible due to dermal fibrosis, necessitating ultrasound-guided central venous catheterization for venous blood sampling and drug administration.

**Figure 2:**
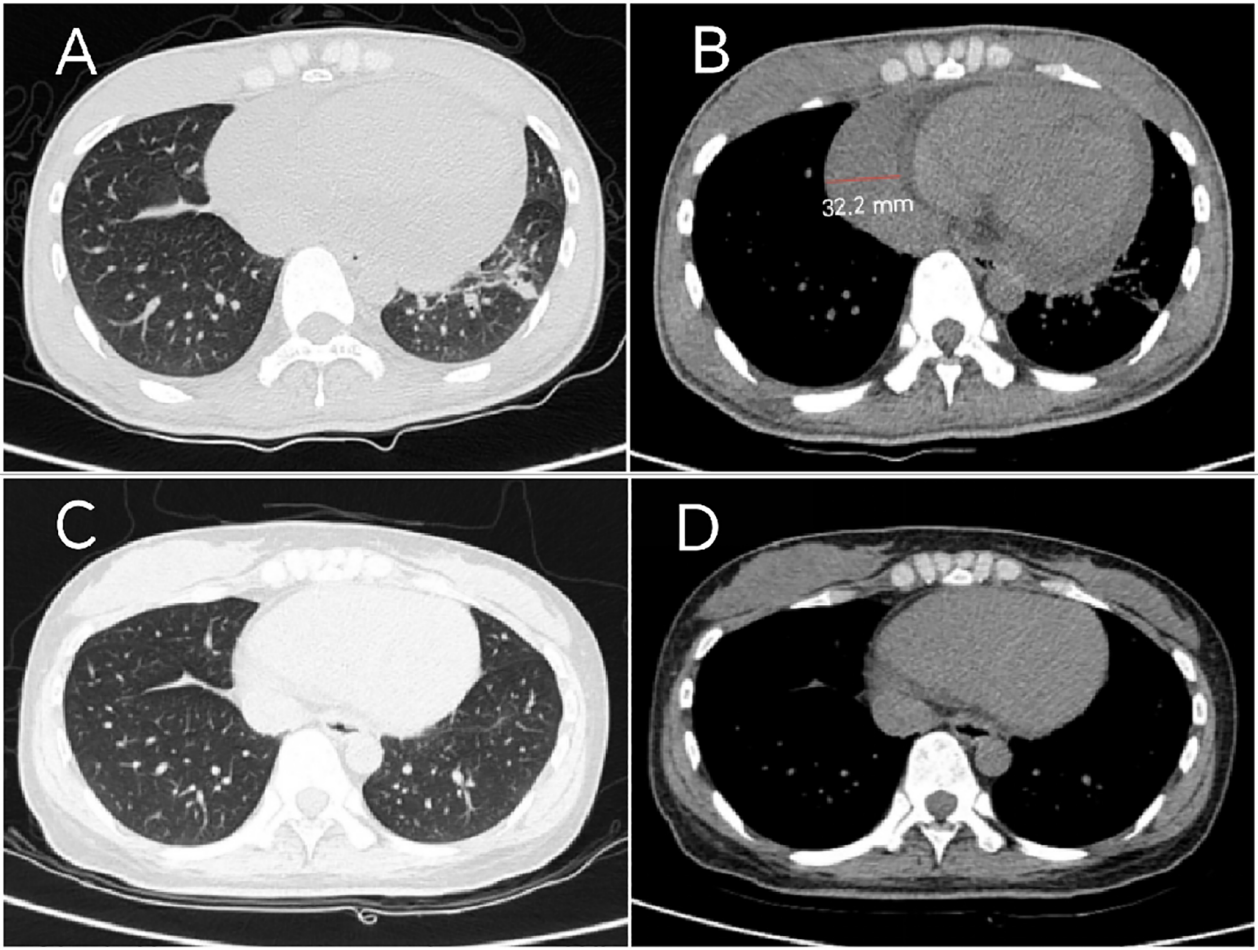
Serial Chest CT findings: **(A)** Baseline HRCT (March 2024) demonstrates bilateral lower lobe slight ground-glass opacities, with left lower lobe reticulation, corresponding to early pulmonary fibrosis. **(B)** Mediastinal window demonstrates moderate pericardial effusion with maximum diameter measuring 32.2 mm. **(C)** Follow-up HRCT (March 2025) reveals reduction in ground-glass opacities with complete resolution of left lower lobe lesions. **(D)** Mediastinal window confirms complete pericardial effusion resolution.

**Figure 3:**
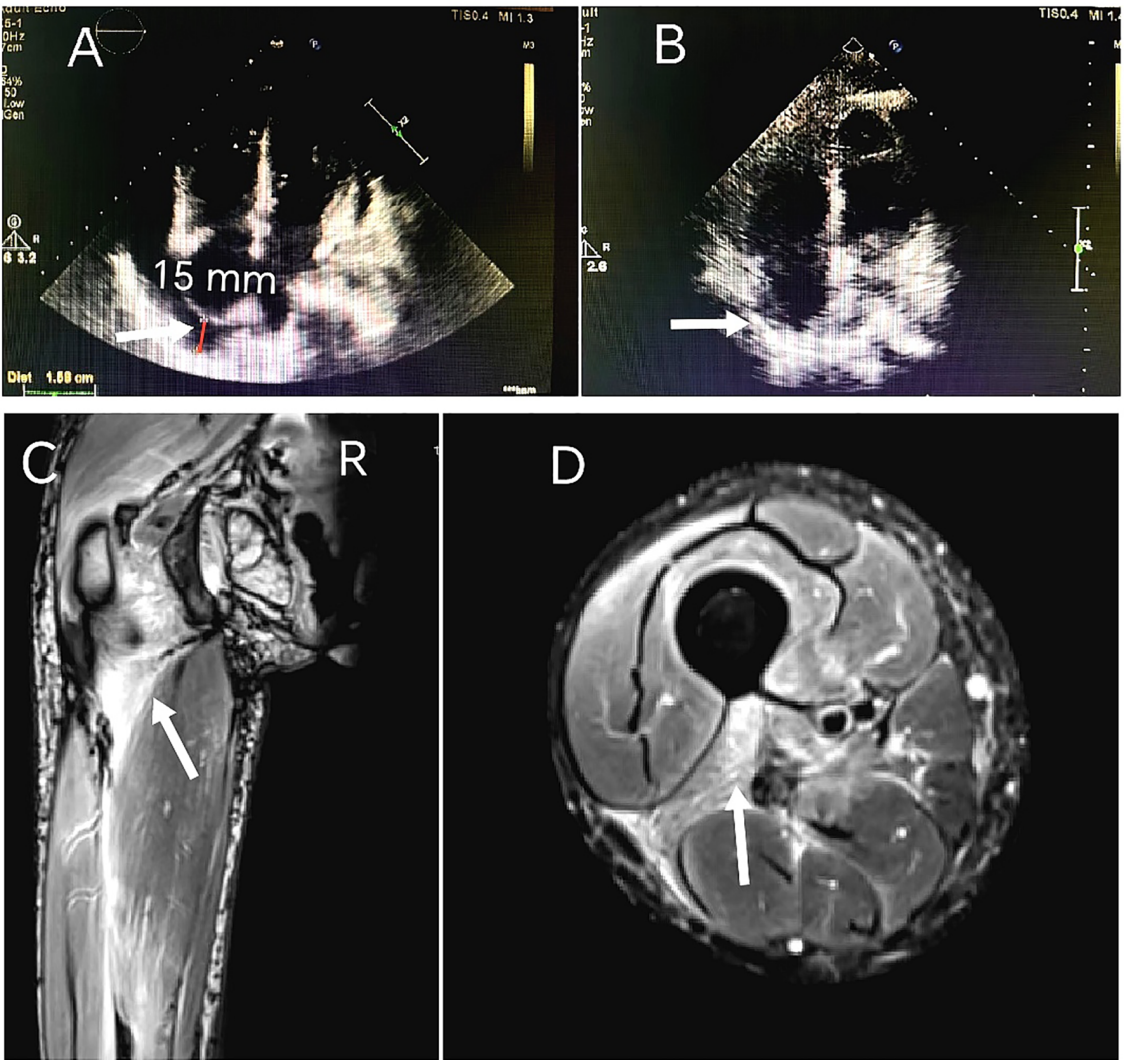
Serial transthoracic echocardiography findings: **(A)** March 2024 demonstrates circumferential pericardial effusion measuring 15 mm in maximal diastolic dimension, quantified using M-mode through the right ventricular free wall. **(B)** 8-month follow-up study (November 2024) reveals complete pericardial effusion resolution. MRI on admission: **(C)** Coronal T2-weighted fat-suppressed imaging of the right thigh reveals multifocal hyperintensity along the femoral greater trochanter and within the intermuscular spaces between the quadriceps femoris muscle groups, consistent with inflammatory fasciitis. **(D)** Axial imaging at the mid-thigh level demonstrates diffuse intramuscular edema.

Laboratory tests revealed elevated levels of creatine kinase (CK 923 U/L; reference range 40-200), lactate dehydrogenase (LDH 396 U/L;120-250), and serum interleukin-6 (IL-6 172.4 pg/mL; <7). Cardiac biomarkers showed mild elevation of brain natriuretic peptide (BNP 205 pg/mL; <125) and troponin I (0.147 ng/mL; <0.014). Inflammatory markers included C-reactive protein (CRP 52 mg/L; <6) and erythrocyte sedimentation rate (ESR 38 mm/h; <20). Myositis-specific autoantibodies (MSAs) were negative. The T-cell test for tuberculosis infection (T-SPOT.TB) was positive, with negative hepatitis B serology and tumor marker profiles. Imaging studies:high-resolution computed tomography (HRCT) of the chest demonstrated mild interstitial fibrosis (**Figure 2A**) despite preserved forced vital capacity (FVC). Cardiac evaluation via HRCT (**Figure 2B**) and echocardiography (**Figure 3A**) identified moderate pericardial effusion and left ventricular dyssynchrony. Musculoskeletal magnetic resonance imaging (MRI) revealed multifocal edema in the right thigh musculature and periarticular hip regions, associated with fascial enhancement (**Figure 3C**). Axial imaging at the mid-thigh level demonstrates diffuse intramuscular edema (**Figure 3D**). Electromyography (EMG) showed no electrophysiological evidence of myopathy. Gadolinium-enhanced cardiac MRI was aborted due to inability to establish intravenous access secondary to diffuse dermal fibrosis. Muscle biopsy and pericardial fluid cytology was unobtainable due to the decline of invasive diagnostic interventions.

**Figure 4:**
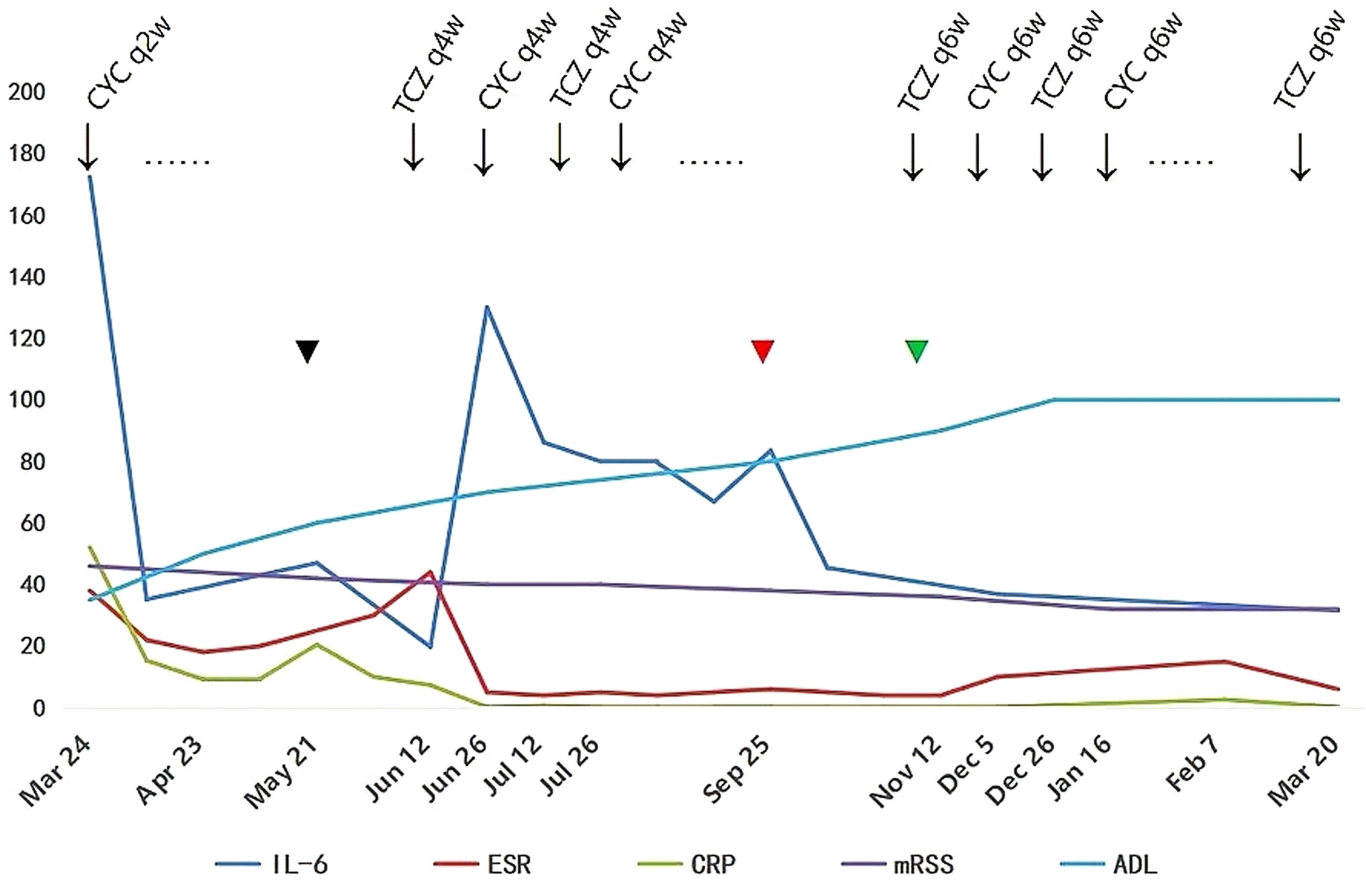
Longitudinal changes in serum interleukin-6 (IL-6), erythrocyte sedimentation rate (ESR), C-reactive protein (CRP), modified Rodnan skin score (mRSS), and Activities of Daily Living (ADL) score during therapy (March 2024 - March 2025). Key events: Initial cyclophosphamide (CYC) every 2 weeks (q2w); 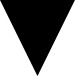 (Black) pseudomonas aeruginosa infection (May 2024) and pericardial effusion unresolved: Added tocilizumab (TCZ) every 4 weeks (q4w) + extended CYC to q4w; 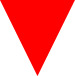 (Red) peripheral venous access restoration (Sep 2024); 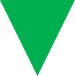 (Green) complete pericardial effusion resolution (Nov 2024):TCZ and CYC interval extended to every 6 weeks (q6w).

A diagnosis of SSc with cardiac and musculoskeletal involvement was established. Given the absence of MSAs, lack of significant CK elevation, and normal EMG findings, the musculoskeletal manifestations were determined to be attributable to SSc rather than overlap polymyositis. Comprehensive evaluation revealed no evidence of TB or malignant neoplasms. The initial therapeutic regimen included intravenous methylprednisolone 40 mg daily for 7 days, transitioning to oral prednisone 50 mg daily, intravenous CYC 400 mg every 2 weeks (q2w), oral MTX 10 mg weekly and nifedipine 10 mg twice daily. After two months of combined CYC-corticosteroid therapy, clinical improvement was observed in myalgia, arthralgia, and dyspnea, accompanied by modest regression of cutaneous sclerosis (mRSS 42); however, pericardial effusion remained unchanged. Laboratory tests showed CK 176 U/L, LDH 246 U/L, IL-6 47.0 pg/mL, CRP 9.5 mg/L and ESR 20.0 mm/h. In May 2024, the patient developed a Pseudomonas aeruginosa-infected cutaneous ulcer (2×2 cm) on the right elbow, successfully treated with levofloxacin 500 mg daily after clindamycin failure. Therapeutic escalation was initiated in June 2024 with the implementation of a phased 4-week therapeutic cycle was implemented, consisting of TCZ 8 mg/kg administered at week 0, followed by CYC 600 mg at week 2, with each agent repeating every 4 weeks (q4w), alongside corticosteroid tapering to prednisone 15 mg daily. Following initial TCZ administration, IL-6 level rose to 130 pg/mL (**Figure 4**). By September 2024, serial imaging demonstrated gradual pericardial effusion resolution and skin softening, restoration of peripheral venous access. Complete pericardial effusion resolution was achieved by November 2024 (**Figure 3B**), prompting adjustment to a phased 6-week therapeutic cycle:TCZ 8 mg/kg administered at Week 0, followed by CYC 600 mg at Week 3, with each agent repeated every 6 weeks (q6w). Concurrently, prednisone was further tapered to 5 mg daily.

At the March 2025 follow-up (main treatment timeline: **Figure 4**), the patient achieved full functional independence with an ADL score of 100/100. Clinical improvement was evidenced by reduced cutaneous sclerosis (mRSS 32 from baseline 46), and weight restoration to 52 kg from nadir 42 kg. Laboratory tests showed CK, BNP,LDH, ESR, and CRP were within normal limits, serum IL-6 31.5 pg/mL (baseline 172.4). HRCT revealed reduction in ground-glass opacities with complete resolution of left lower lobe lesions (**Figure 2C**) and mediastinal window confirms complete pericardial effusion resolution (**Figure 2D**). Therapeutic tolerance was excellent, and no treatment-related adverse events were documented.

## 3 Discussion

The burden of SSc disease is high, particularly for patients with visceral involvement, who often experience a poorer quality of life and face a significantly higher risk of mortality (6). This risk is further exacerbated in those with rapidly progressive SSc (4). Cardiovascular complications occur in up to one-third of patients with SSc (7), and the presence of overt clinical signs of cardiac disease is associated with a poor prognosis (8). Pericardial involvement is common in SSc patients, but pericardial effusion should be managed conservatively, as it is considered a sign of potentially worsening disease (9). Notably, even asymptomatic large pericardial effusions portend adverse outcomes (10).

In this case, we describe a young female patient with rapidly progressive SSc with cardiac involvement who was successfully treated with a combination of CYC and TCZ. At the molecular level, SSc is a heterogeneous disease associated with T cells, B cells, and profibrotic cytokines (11). T cells and B cells can promote fibrosis through complex mechanisms (12). IL-6 plays a role in the pathogenesis of SSc. Desallais et al. (13) demonstrated in a mouse model study that serum and skin IL-6 levels are significantly elevated in patients with early SSc. Furthermore, Sato et al. (14) reported that serum IL-6 levels closely correlate with the severity of skin thickening in SSc, suggesting that IL-6 may serve as a potential serological biomarker for skin fibrosis in this disease. This patient exhibited abnormally elevated levels of B cells and IL-6. CYC, as an alkylating agent, remains a cornerstone therapy for severe SSc, particularly in patients with early, progressive disease (1, 2, 4, 12). High-dose glucocorticoids combined with CYC have demonstrated efficacy in treating systemic lupus erythematosus-associated pericardial effusion (15). The immunomodulatory effects of CYC arise from cytotoxic intermediates that trigger a cascade of events, including selective depletion of regulatory T cells and enhancement of effector T cell function, alongside systemic immune modulation (16). However, in this case, the patient’s pericardial effusion did not improve with initial glucocorticoid and CYC therapy. Subsequently, she developed cutaneous ulceration with superimposed infection, necessitating an urgent shift in therapeutic strategy.

The detection of significantly elevated serum IL-6 levels (172.42 pg/mL; 24-fold above upper limit of normal) on cytokine profiling mechanistically justified therapeutic intensification, culminating in a combined immunomodulatory approach with CYC and TCZ. As an IL-6 receptor inhibitor, TCZ suppresses IL-6-mediated inflammatory pathways and has shown promise in managing SSc-related cutaneous and pulmonary manifestations (3, 5, 17). The current rheumatology consensus supports TCZ use in SSc patients with interstitial lung disease, particularly those positive for anti-topoisomerase antibodies, experiencing disease progression, or intolerant to CYC due to adverse effects or treatment failure (3). Nevertheless, the pathophysiological role of IL-6 in SSc-associated cardiovascular complications remains poorly defined. Preclinical evidence from Li et al. (18) highlights a novel fibroblast-derived IL-6 mechanism: in murine myocardial infarction models, IL-6 expression was upregulated in cardiac fibroblasts and myocardium, while fibroblast-specific IL-6 knockdown significantly attenuated cardiac fibrosis. Clinically, Ishizaki et al. (19) reported the first successful use of TCZ (8 mg/kg q4w) in a 44-year-old woman with SSc-related myocardial fibrosis manifesting as multifocal premature ventricular contractions. Additionally, Funauchi et al. (20) described a 13-year-onset SSc with suspected myocardial fibrosis (characterized by multifocal ventricular premature contractions and reduced left ventricular ejection fraction to 50%) who achieved clinical improvement with subcutaneous TCZ (162 mg q2w) monotherapy without significant adverse events. Notably, prior cases reported TCZ monotherapy for arrhythmias or systolic dysfunction without pericardial effusion. The successful resolution of pericardial effusion in our patient via combined CYC-TCZ represents a novel therapeutic approach for this complication.

However, the combination of CYC and TCZ remains understudied. To balance infection risk and therapeutic efficacy, phased 4-week cycle was implemented: TCZ at Week 0, CYC at Week 2, both agents repeated q4w. Remarkably, the patient’s pericardial effusion was completely resolved and IL-6 levels were significantly reduced (172.4 → 31.5 pg/mL) following this combined therapy. We noticed that IL-6 levels peaked at 130 pg/mL following initial TCZ administration and then gradually declined (**Figure 4**). This phenomenon aligns with the mechanistic findings reported by Nishimoto et al. (21), which demonstrate that serum IL-6 elevation occurs in rheumatoid arthritis patients receiving TCZ, as TCZ-bound soluble IL-6 receptor impedes IL-6 clearance from circulation. In this patient, the subsequent decline in IL-6 correlated with reductions in CRP and ESR levels, likely attributable to resolution of underlying inflammation and extended TCZ dosing interval q6w.

This case report illustrates the successful application of CYC-TCZ combination therapy in a patient with rapidly progressive, refractory SSc. It suggests that dual-pathway targeting—combining immunosuppression (CYC) with anti-inflammatory action (TCZ)—may exert additive therapeutic effects in SSc. Unlike traditional therapies targeting isolated pathways, this strategy concurrently addresses fibrotic progression and inflammatory cascades. Mechanistically, TCZ directly inhibits IL-6-mediated inflammation, thereby complementing the broad immunosuppressive effects of CYC. Synergistically, this approach targets both fibrosis and inflammation, potentially disrupting the self-perpetuating cycle of SSc pathogenesis.

Despite its therapeutic efficacy, CYC carries risks of long-term toxicities such as gastrointestinal complications, infections, respiratory dysfunction, and hematologic/lymphatic system abnormalities (22). In contrast, TCZ demonstrates a more favorable safety profile, with common adverse events limited primarily to infections and cardiovascular complications (17). Given the patient’s concurrent cutaneous infection, CYC dosing was extended to q4w. This adjustment aimed to balance immune reconstitution dynamics following CYC-induced lymphodepletion: excessively short intervals risk depleting activated natural killer cells and cytotoxic T lymphocytes, whereas too long an interval may allow cells to acquire drug resistance (16). Notably, despite infection complications during initial corticosteroid-CYC therapy, subsequent dose optimization was implemented through a phased 4-week cycle. This individualized approach resulted in: (i) no new adverse events; (ii) restoration of peripheral venous access by Month 3; and (iii) multi-system improvements at Month 6: mRSS (46 → 32), joint mobility, ADL score (35 → 100), and pericardial effusion resolution. Given sustained symptomatic resolution and the need to mitigate adverse effect risks, the regimen was transitioned to a 6-week cycle to optimize therapeutic exposure while minimizing cumulative toxicity.

These findings underscore the potential of CYC-TCZ combination therapy to improve clinical outcomes in SSc. Key limitations warrant consideration: First, inability to confirm myocardial fibrosis via cardiac MRI due to contraindications for intravenous contrast administration (severe cutaneous sclerosis); Second, histopathological validation (e.g., muscle biopsy or pericardial fluid analysis) was not obtained due to the patient’s explicit decline of invasive diagnostic interventions, thereby constraining precise etiopathogenetic analysis. Third, the generalizability of this therapeutic approach to broader SSc populations remains uncertain. Future research should prioritize prospective studies evaluating the long-term efficacy-toxicity profile of this combination, especially in early progressive SSc subtypes. Therapeutic individualization—tailoring regimens to disease trajectory and comorbidity burden—remains paramount for optimizing risk-benefit ratios.

This case demonstrates the feasibility of CYC-TCZ therapy in refractory SSc with cardiac involvement, supporting the hypothesis that multi-target strategies may synergistically attenuate inflammatory and fibrotic pathways in this complex disease. Mechanistically, CYC’s broad immunosuppression coupled with TCZ’s IL-6 pathway blockade may disrupt the self-amplifying loop of SSc pathogenesis.

## Data Availability

The original contributions presented in the study are included in the article/Supplementary Material. Further inquiries can be directed to the corresponding author.
